# The in-silico feasibility of dose escalated, hypofractionated radiotherapy for rectal cancer

**DOI:** 10.1016/j.ctro.2022.06.003

**Published:** 2022-06-11

**Authors:** Lynsey Devlin, Laura Grocutt, Bianca Hunter, Hiwot Chemu, Aileen Duffton, Alec McDonald, Nicholas Macleod, Philip McLoone, Sean M. O'Cathail

**Affiliations:** a Department of Radiotherapy, The Beatson West of Scotland Cancer Centre, Glasgow, United Kingdom; b CRUK RadNet Glasgow, University of Glasgow, Glasgow, United Kingdom; c Department of Radiotherapy Physics, The Beatson West of Scotland Cancer Centre, Glasgow, United Kingdom; d Department of Clinical Oncology, The Beatson West of Scotland Cancer Centre, Glasgow, United Kingdom; e Institute of Health & Wellbeing, University of Glasgow, Glasgow, United Kingdom; f Institute of Cancer Sciences, University of Glasgow, Glasgow, United Kingdom

## Abstract

**Background and purpose:**

Short course radiotherapy (SCRT) has a low biological prescription dose. Rectal cancer has a dose response relationship and moderate α/β ratio (∼5). We hypothesise hypofractionated dose escalation has radiobiological advantages. We assessed in-silico dose escalation to the primary tumour using a simultaneous integrated boost (SIB) technique.

**Materials and methods:**

Patients who had received 25 Gy/5# were enrolled. GTV was macroscopic tumour including lumen. CTVA was GTV + 10 mm. CTVB included elective nodes. PTV_Low was created from CTVF (CTVA + CTVB) + 7 mm. PTV_High (SIB) was GTV + 5 mm margin. OAR were as per RTOG guidelines. Each patient had 4 plans created at increasing dose levels (27.5 Gy, 30 Gy, 32.5 Gy and 35 Gy) to PTV_High. PTV_Low was 25 Gy/5#.

5 test plans were created for each patient in Eclipse™ v15.5 and consisted of 2 VMAT full arcs (6 MV), Varian Truebeam (2.7). Planning objectives were set in the Photon optimiser (PO) and recalculated using Acuros v15.5. *A priori* feasibility was defined as 90% of plans achieving the planning objectives at 32.5 Gy dose level (EqD2 53.4 Gy).

**Results:**

20 SCRT patients median age 70, F (n = 5), M (n = 15). Rectum level; low (n = 12), mid (n = 3) and upper (n = 5). 100 plans were analysed. Mean volume of PTV_High was 130 cm^3^ (SD 81.5) and PTV_Low 769.6 cm^3^ (SD 241.1). 100% plans complied with mandatory planning dose metrics for each structure at the 25 Gy/5# plan and each dose level.

**Conclusion:**

Hypofractionated dose escalation to the primary tumour up to 35 Gy/5# is technically feasible in rectal cancer radiotherapy.

## Highlights

• In silico dose escalation to the primary tumour up to 35 Gy/5# in SCRT for rectal cancer is feasible.
• Mandatory study planning objectives were adhered to for all patients up to 35 Gy.
• Dose to organs at risk did not increase at plan dose increment levels (27.5 Gy, 30 Gy, 32.5 Gy and 35 Gy).

## Introduction

Radiotherapy plays a key role in the management of rectal cancer. In the locally advanced setting, neoadjuvant radiation decreases local recurrence, through a combination of tumour regression and pelvic sterilisation of microscopic disease [1]. Numerous, large, high quality randomised controlled trials demonstrate this can be achieved by either pre-operative 45–50.4 Gy /25–28# with concurrent fluoropyrimidines [2], [3] long course chemoradiotherapy (LCCRT) or 25 Gy/5# short course radiotherapy (SCRT) [4], [5]. Direct comparison shows no long-term superiority of either approach [6], [7]. Despite this equipoise, LCCRT dominates the landscape as the *de facto* standard of care [8], [9]. The original schedule for short course radiotherapy (SCRT) involves surgery within 7–10 days of radiotherapy completion, presenting logistical challenges. Additionally, tumours have a low pathological complete response (pCR) rate of ∼ 1% [6]. Recently, the phase 3 non-inferiority Stockholm III trial [10] demonstrated SCRT plus a 4–8 weeks delay, is an equivalent neoadjuvant approach in terms of local control. The pCR increased to 10.4%, from 0.3% in the SCRT plus immediate surgery arm, closer to that seen in LCCRT. The use of SCRT is further reinvigorated by the practice changing sequential administration of neoadjuvant chemotherapy [11], [12].

SCRT has significant advantages for patients and health systems over LCCRT. Patients have less hospital visits and it is cheaper to deliver [13]. US cost-effectiveness studies comparing SCRT and LCCRT favour SCRT [14], [15], [16].

The demonstrable equivalence in clinical outcomes of a moderately hypofractionated regime (25 Gy/5#) and standard fractionation regime (50 Gy/25#) bears further radiobiological interrogation. The relationship between fraction size and tumour/tissue response is well described by the α/β value in the linear quadratic model of fractionation sensitivity [17]. Low α/β values signify greater sensitivity to fraction size than higher α/β values. The outlined prescriptions are not *iso*-effective unless the α/β value of rectal adenocarcinoma is moderate to low, this has been estimated to be ∼ 5 Gy [18].

Finally, there exists a dose–response relationship in rectal adenocarcinoma. Higher overall tumour dose results in improved tumour regression [19]. The dose response curve found to be steepest between 50 and 70 Gy in standard fractionation, suggesting that rectal cancers are routinely under dosed in radiobiological terms for maximal response. Despite this dose escalation trials in LCCRT have had mixed results, the RECTAL-BOOST study reporting no increase in pCR rates using a sequential boost technique [20]. Encouragingly a systematic review of inverse planned techniques found improved pCR rates with doses greater than 54 Gy [21]. This supports the trend towards organ preservation strategies, with potential in both the LCCRT and SCRT settings for dose escalation to avoid surgical morbidity, a permanent stoma and improve patient’s quality of life (QOL) [22].

Given the low biological dose of SCRT (25 Gy/5#), the existence of a dose response and the moderate α/β ratio (∼5) of rectal cancer, we hypothesise that hypofractionated dose escalation would have significant radiobiological advantages. We aimed to assess the in-silico feasibility of dose escalation to the primary tumour using a simultaneous integrated boost (SIB) technique planned for 5 fractions. Dose to the SIB volume was increased by 2.5 Gy increments whilst conserving organs at risk (OAR). Plans were created over five dose levels as shown in Table 1. Dose levels were 25 Gy/5# (level 1), 27.5 Gy/5# (level 2), 30 Gy/5# (level 3), 32.5 Gy/5# (level 4) and 35 Gy/5# (level 5).

**Table 1:** The EqD2 of dose levels 1–5 for α/β values of 10, 5 and 3.

**Dose level**	**Dose/fractionation**	**EqD2** **α/β 10**	**EqD2** **α/β 5**	**EqD2** **α/β 3**
**1**	25 Gy/5	31.3 Gy	35.7 Gy	40 Gy
**2**	27.5 Gy/5	35.5 Gy	41.2 Gy	46.8 Gy
**3**	30 Gy/5	40 Gy	47.1 Gy	54 Gy
**4**	32.5 Gy/5	44.7 Gy	53.4 Gy	61.8 Gy
**5**	35 Gy/5	49.6 Gy	60 Gy	70 Gy

**Table 2:** Study patient characteristics (median values with interquartile range in brackets).

**Patient characteristics**	
No. of patients	20
Age years (median [IQR])	71.5 [63.75–75]
Female/Male	F = 5 M = 15
T stage (n)	
T1	1
T2	4
T3	11
T4	4
N0/N1	14/6
Rectum level (n)	
lower/mid/upper	12/3/5
PTV_low (cm^3^)	740 (610.5–836.8)
PTV_high (cm^3^)	110.3 (76.7–149.6)
GTV (cm^3^)	40.5 (30.8–71)
Small Bowel (cm^3^)	153.9 (97.5–276.2)
Bladder (cm^3^)	164.7 (121.4–364)
Bowel cavity (cm^3^)	967.9 (549.7–1088.1)
Large Bowel (cm^3^)	19.31 (9.7–34.4)
Rectal diameter (cm)	3.7 (3–4.3)
Tumour length (cm)	4.8 (3.5–6.3)

## Methods

Operable rectal cancer patients who previously received neoadjuvant SCRT (25 Gy/5#) were included. Palliative/local control patients were excluded. Patients were immobilised supine with knee and ankle supports using Prostep (Medizintechnik, GMBH), instructed to empty their bowels/bladder and drink 500mls 30 minutes before scanning. A planning CT scan was acquired using a Phillips Brilliance Big Bore (Phillips Medical Systems, USA) scanner with intravenous contrast, unless contraindicated by renal function. Scan extent including L2/3 to mid-femur with a 2–2.5 mm slice thickness. CT data sets were anonymised using ARIA v15.1 (Varian Medical systems, Palo Alto) to create a test patient library. Institutional approvals were obtained.

### Delineation of structures

The GTV was the macroscopic primary tumour including the circumferential rectal lumen. The diagnostic MRI scan was used alongside the planning CT to aid delineation. The CTVA included the GTV + 1 cm margin. The CTVB included the mesorectum, internal iliac, presacral and obturator lymph nodes, outlined in accordance with the UK rectal IMRT guidance [23]. Involved/suspicious nodes were not boosted. CTVF was summation of CTVA and CTVB. PTV_Low was created from CTVF by adding a 7 mm margin and PTV_High (SIB) from the GTV by adding 5 mm [24], [25].

OAR were delineated as per RTOG definitions [26], bladder including the wall and lumen, small bowel loops and bowel cavity. Bowel cavity was defined as most inferior small or large bowel loop, or above the rectum, whichever was most inferior. The small bowel loops and bowel cavity were contoured 5 cm above PTV_Low. The left and right femoral heads were contoured from femoral head to lesser trochanters. Large bowel was defined as 2 cm outside of PTV_High with an inferior limit of levator-ani muscles. Plans were not optimised using the large bowel structure, this structure was used to record dose metrics at D_1cc_ < 30 Gy, D_0.5cc_ < 32 Gy [27] for plan dose levels 32.5 Gy and 35 Gy.

All structures were delineated by a single Clinical Oncologist (CO), to reduce inter-observer variation, and independently checked by another CO.

### Treatment planning system and dose calculation

Treatment plans were created and checked by two experienced planners independently using the Eclipse™ treatment planning system (TPS) PO v15.5 and Acuros v15.5 [Varian Medical Systems, Palo Alto, Ca, USA] for final calculation on a grid size of 2 mm. Volumetric modulated arc therapy (VMAT) plans were generated using 6MV photons at dose-rate of 600 MU/min. The maximum leaf speed was 2.5 cm/sec with a maximum gantry speed of 6°/sec, arc control points every 2°. Plans consisted of two full coplanar arcs, collimator 30° clockwise and 330° counter-clockwise. Collimator jaw tracking was used for each plan. Dose was prescribed to the median target dose with criteria according to ICRU83 [28]. For the level 1 plans this was PTV_Low and for dose levels (2–5) PTV_High. Plans were not actively optimised to control dose from PTV_High into PTV_Low, with ‘dose spill’ allowed. This was deemed beneficial due to differential motion of the targets and the absence of a CTV for PTV_High. Optimisation objectives for PTV and OAR are detailed in Supplementary Table 1.

### Planning aims and optimisation

**Fig. 1:**
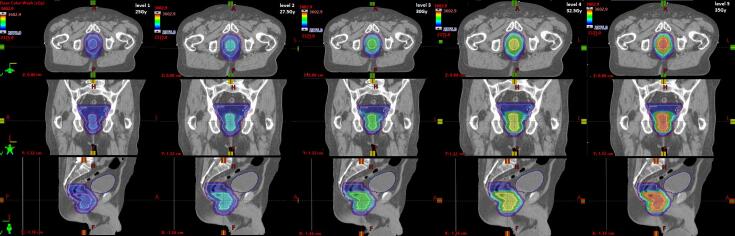
Example of study patient planning CT showing dose colour wash at baseline 25 Gy/5# (level 1) and dose levels 2–5 (27.5 Gy, 30 Gy, 32.5 Gy and 35 Gy) with increasing dose to PTV_High.

For each test patient, five treatment plans were produced; a 25 Gy/5# plan (level 1) and at each of the 2.5 Gy incremental dose levels (2–5) to the SIB volume PTV_High. The dose prescribed to PTV_Low was 25 Gy/5# for level 1 and PTV_High in dose levels (2–5) was prescribed 27.5 Gy/5#, 30 Gy/5#, 32.5Gy/5# and 35 Gy/5# respectively (Fig. 1.).

All plans were optimised within Eclipse™ optimization workspace where objectives were manually defined. Optimisation objectives were applied to generate dose volume histogram (DVH) estimates. PTV objectives took priority (100%) over OAR to ensure optimal PTV coverage. The bowel and bladder were assigned priorities of 60% and 40% respectively. Given that femoral heads receive minimal dose, these were not assigned specific objectives. Following evaluation, PTV or OAR parameters were modified if necessary to achieve the optimal plan. Plans were not re normalised.

### Plan evaluation

Individual plan dose distributions were assessed by a CO to confirm clinical acceptability and dose-volume parameters were compared. Plan acceptability was defined as clinically acceptable, minor where optimal constraints were exceeded and major where mandatory constraints were exceeded. Feasibility was defined as 90% of plans achieving the planning objectives at 32.5 Gy dose level 4 (EqD2 53.4 Gy). Each of the five plans were quantitatively compared, using DVH analysis of the PTV and OAR objectives (Supplementary Table 1.).

### CBCT imaging evaluation

Study patient’s 3D cone-beam CT (CBCT) images were available for all patients, previously treated with 25 Gy/5#. Patients planning CT were retrospectively registered to daily CBCT images using a standardised region of interest by a radiation therapist. The study structure set was used to verify anatomical coverage on each CBCT. Visually assessed by checking if the CTV was encompassed by the PTV_Low and the GTV by PTV_High. To determine if delivery of the dose escalated plans was feasible, CBCT images were categorised as acceptable coverage, a partial miss or geographical miss by a CO.

### Plan quality metrics

Quality indices were used to assess PTV_High and PTV_Low for all plans. The conformity index (CI_RTOG_) assesses the target volume coverage and normal tissue sparing. In this study, the CI was calculated using the formula CI = Volume of reference isodose/target volume [29]. The homogeneity index (HI) analyses the uniformity of the dose distribution in the target volume and was calculated using formula HI = D5%/D95% [30]. Results closest to 1 represent optimal conformality and homogenous dose. Finally, the dose fall off index (DFI) assesses the high-dose fall-off (107% of prescribed dose) from PTV_High into PTV_Low. DFI was calculated for the PTV_High (SIB) volume using the formula DFI = Volume of 107%/target volume, adapted from RT0G915 [31]. DFI was calculated for PTV_High, PTV_High + 5 mm margin and PTV_High + 10 mm margin.

Plan deliverability was assessed via pre-treatment quality assurance (QA) measurements across all plans. Planned doses were delivered by a Varian TrueBeam™ linear accelerator, compared to delivered doses using phantom MapCheck2 with 2D global gamma-analysis criteria of 3%/3mm. Deliverability and plan complexity was assessed using the dose-rate and gantry speed for each control point. RadCalc v6.3 independently checked monitor units (MU) alongside plan complexity parameters Average Leaf Pair Openings (ALPO) and Modulation Factors (MF).

### Statistical analysis

We summarised continuous variables as means (standard deviations) or as medians (inter-quartile ranges). Counts of plans achieving mandatory and optimal constraints were recorded. Dose metrics were extracted from DVH’s for all plans, exported in 0.05 Gy resolution from Eclipse v15.5 (Varian Medical Systems, Palo Alto, CA). Statistical analysis was performed using Stata v14.

## Results

Twenty patients were included in the analysis, patient characteristics and structure volumes are reported in Table 1. One hundred treatment plans were analysed.

**Fig. 2:**
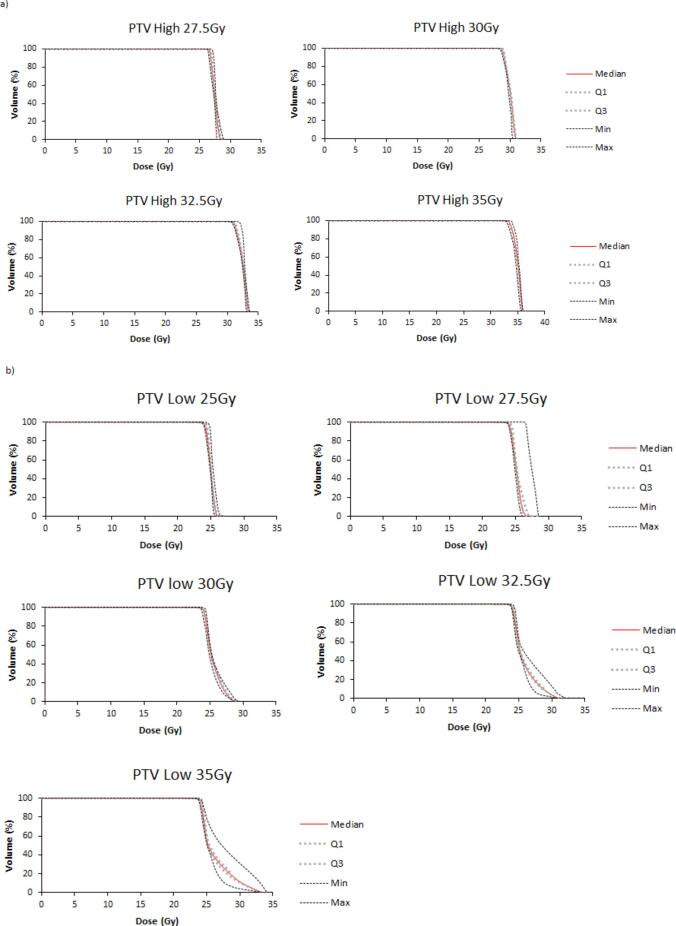
Population dose volume histograms for (a) PTV_High and (b) PTV_Low showing the median, interquartile range and min and max values.

**Fig. 3:**
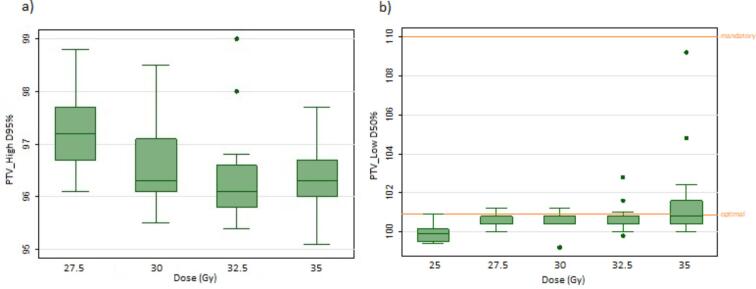
Boxplots showing dose constraints at dose levels (27.5 Gy, 30 Gy, 32.5 Gy and 35 Gy) for structures (a) PTV_high D95% > 95% and (b) PTV_low D50% = 99–101% (mandatory 〈1 1 0). The band represents the median value, the box the first and third quartile. The whiskers show variation above and below this with outliers as dots.

All plans (100%) achieved the planning objectives at the 25 Gy/5# (level 1) and each of the increasing dose levels 2–5. Dose metrics were generally consistent across the dose levels (Supplementary Table 2). Fig. 2 shows population DVH for PTV_Low and PTV_High with DVH for all other structures in Supplementary Fig. 1. For the PTV_Low D50%, all plans achieved the mandatory constraint resulting in zero major deviations. The optimal constraint was exceeded by 7 plans (7%) categorised as minor deviations (30 Gy n = 1, 32.5 Gy n = 2, 35 Gy n = 4), for 4/20 patients (Fig. 3).

Minimal increase in OAR dose across all dose levels was observed. Population DVH’s for small bowel, bladder, large bowel, right and left femoral heads can be found in supplementary Fig. 1. For large bowel, mean D_1cc_ for 32.5 Gy and 35 Gy plans were 27.0 Gy (SD 414.9), 29.2 Gy (SD 308.9) and for D_0.5cc_ was 27.8.6 Gy (SD 395.4) and 30.2 Gy (SD 270.7) respectively. The D_1cc_ was exceeded in only 1 plan (5%) at 32.5 Gy and 7 plans (35%) at 35 Gy. The D_0.5cc_ was exceeded in 4 plans (20%) at 32.5 Gy and 4 plans (20%) at 35 Gy. As plans were not optimised with this constraint, these were deemed to be minimal breaches with a maximum value of 1.5 Gy.

CO plan evaluation of daily CBCT (n = 100) defined coverage of the target volumes as acceptable for 84 fractions (84%), partial miss for 11 fractions (11%) and geographical miss for 5 fractions (5%). Per patient occurring for 12/20, 7/20 and 1/20 respectively. Observed reasons for poor target coverage were due to patient position or poor bladder/rectal preparation on planning CT/CBCT.

**Fig. 4:**
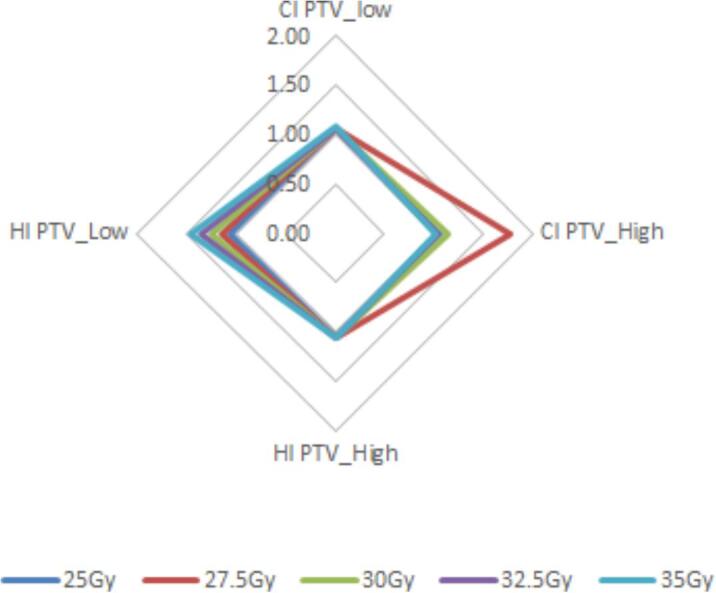
Radar diagram showing the mean HI for PTV_Low and PTV_High. The HI for PTV_Low increases up the dose levels due to the effect of dose spill from PTV_High into PTV_Low. The CI value is greatest for the 27.5 Gy (dose level 2) due to the small dose increase from PTV_Low at 25 Gy and the SIB volume being 27.5 Gy (values closest to 1 represent ideal conformality and homogenous dose).

The mean CI and HI is shown in Fig. 4. HI was consistent across all dose levels representing homogenous dose, increasing for PTV_Low at 35 Gy (level 5) due to dose spill from PTV_High. The CI for PTV_High was highest at 27.5 Gy (level 2), due to the small difference of 2.5 Gy dose between PTV_Low and PTV_High. Results for DFI analysis of PTV_High show the fall off from PTV_High into PTV_Low to be gradual. There was a reduction in 107% dose at the 5 mm boundary of 40.6%, 41.6%, 41.5%, 41.5% and at 10 mm of 63.2%, 63.7%, 63.5% and 63.5%. At 10 mm there was minimal 107% dose, an expected effect due to allowing dose spill during optimisation. Found to be within 1% across all dose levels.

Plan complexity was assessed using the metrics shown in Supplementary Table 3. The mean ALPO decreases and indicates greater plan complexity. The MF factor remains relatively unchanged and indicates similar MLC modulation across all 5 dose level plans. The greater plan complexity is due to the increase in total MU, increase in average dose-rate and decrease in average gantry speed. Patient-specific QA performed for each plan suggests all plans were comparable in terms of plan calculation accuracy and deliverability.

## Discussion

Here we define *in-silico* feasibility of dose escalation to the primary tumour up to 35 Gy/5# in SCRT for rectal cancer. We found 100% of plans met our mandatory planning objectives at all dose levels up to 35 Gy. Our results demonstrate the feasibility of hypofractionated dose escalation, with all plans achieving the planning objectives up to 32.5 Gy (EqD2 53.4 Gy), the pre-defined study threshold.

To our knowledge this is the first *in-silico* study investigating dose escalation up to 35 Gy in SCRT for rectal cancer using a VMAT technique with a SIB volume.

VMAT is documented to be the best technique for SIB planning whilst sparing dose to OAR in LCCRT [32], [33], [34]. Our technique uses two arcs, shown to be advantageous, due to target complexity requirements to achieve optimum dose distributions [32].

Automated planning (AP) solutions such as RapidPlan have shown benefit in OAR sparing whilst planning SIB volumes in LCCRT [35]. Our results show planning objectives can be achieved with manual optimisation, where mandatory dose objectives were met. On an individual level, some cases failed optimal objectives at the higher dose levels. We plan to build a RapidPlan model using the 100 study plans to investigate if AP would further improve dose metrics for SCRT dose escalation.

Our optimisation process did not actively control dose, that is, we allowed dose to spill from PTV_High into PTV_Low. Due to the absence of a CTV at the SIB volume this was a desired effect, utilising the gradual dose fall off. In rectal cancer this is beneficial for feasibility of treatment delivery.

The patient’s in our study represent a range of disease staging and tumour locations in the rectum. Our study was retrospective, therefore we did not optimise patient preparation specifically for this technique. We observed a wide range of bladder volumes, the varying presence of rectal filing/gas, with no limits set on rectal diameter. Prospective protocols would mitigate these issues to ensure planning and treatment image suitability, reduce incidences of partial miss which was 11% in our CBCT analysis. We observe GTV would have been encompassed by the gradual dose fall off. Only one patient was considered a geographical miss and in clinical practice a repeat optimised planning CT scan would have been performed. This study thus represents a ‘worst case’ scenario.

Visualisation of soft tissue on CBCT is poor for rectum. Nevertheless, on all daily CBCT images it was possible to assess coverage of GTV with PTV_High. The described technique, where the GTV includes the rectal lumen and not only tumour, is advantageous. CBCT was sufficient to visualise rectal wall in all directions and to differentiate from close by structures. Improving the localisation of the tumour on CBCT to guide online decision making may allow further refinement and margin reduction [36]. Radiopaque marker insertion in rectal cancer radiotherapy has been shown to be safe and feasible [37], an approach that may work best in SCRT where treatment delivery is over five days with less tumour regression or migration.

The rectum has known motion, found to be greatest in the upper/mid sections and less in the lower section [38]. Safe delivery of this technique requires minimal intrafraction rectal motion, essential when delivering a high dose per fraction using a SIB volume [34]. The advantage of this technique over a sequential or concomitant boost is that the SIB utilises the dose distribution that VMAT planning offers. Therefore, implementation requires considerations to ensure safe delivery, with image guided radiotherapy strategies in line with stereotactic ablative radiotherapy techniques [27].

Dose escalation in SCRT could improve pCR response rates, increase surgical avoidance for patients and reduce morbidity. Toxicity has been found to be similar with doses greater than 60 Gy and greater pCR response rates [21]. The risk of small bowel toxicity appears to be the greatest concern in dose escalation from LCCRT studies [39]. VMAT planning has been shown to have less toxicity in comparison to fixed IMRT fields [40]. However, the effects of acute and late toxicity of hypofractionated dose escalation for rectal cancer are relatively unknown. Our analysis did not consider surrounding organs anal canal or genitalia due to the absence of published equivalent constraints for late toxicity [27]. Dose to anal canal may be important in the organ preservation setting where maintaining bowel function is vital. To establish the safety of hypofractionated dose escalation in rectal cancer we are developing a prospective phase 1 study. Patient reported outcome measures will be collected to establish dose effects on bowel function and QOL.

Currently an ongoing Phase 1 study in China [41] investigates this approach for rectal cancer, using higher doses (30 Gy/5#, 35 Gy/5# and 40 Gy/5#) [42]. The highest proposed dose of 40 Gy/5# would result in a very high biological dose if the α/β ratio proves to be moderate (EqD2 α/β 5 = 74.3 Gy). This is higher than the estimated dose response range for rectal cancers and may be unnecessarily overdosing the tumours [19].

This manuscript has been prepared in accordance with the radiotherapy treatment planning study guidelines (RATING) framework, achieving a score of 95% [43].

## Conclusion

Hypofractionated dose escalation to the primary tumour up to 35 Gy/5# is feasible in SCRT for rectal cancer. All plans met our *a priori* definition of feasibility at 32.5 Gy (EqD2 53.4 Gy). Safety and feasibility of this technique will be investigated in a Phase 1 clinical trial.

## Funding

Funding for research post held by LD is provided by the Beatson Cancer Charity and by CRUK RadNet Glasgow (C16583/A28803).

LG funding is provided by CRUK RadNet Glasgow (C16583/A28803).

SMO’C is a CRUK funded clinical senior lecturer at the Institute of Cancer Sciences (grant number CAN-RES-UK (C7932/A25142).

AD funding is provided by the Beatson Cancer Charity.

Funding for statistical support from PM was provided by the Beatson Cancer Charity.

## Declaration of Competing Interest

The authors declare that they have no known competing financial interests or personal relationships that could have appeared to influence the work reported in this paper.
