# Supplementary material for: The in-silico feasibility of dose escalated, hypofractionated radiotherapy for rectal cancer
Source: Clin Transl Radiat Oncol. 2022 Jun 11;36:24–30. doi: 10.1016/j.ctro.2022.06.003 (PMC9218294; doi:10.1016/j.ctro.2022.06.003)
Supplement: Supplementary data 2 [file mmc2.docx]

**Table 1.** Planning optimisation objectives

|  |  |  |
| --- | --- | --- |
| Structure | Objective | |
| PTV_High (SIB) | D99% | >90% |
|  | D95% | >95% |
|  | D50% | 99-101% |
|  | D2% | <105% |
| PTV_Low | D99% | >90% |
|  | D95% | >95% |
|  | D50% | 99-101%* |
| PTV_Low – PTV_High + 5mm | D107% | <15% |
| Bladder | D45% | <21Gy |
| Small Bowel | D200cc | <20Gy |
|  | D150cc | <22Gy |
|  | D20cc | <25Gy |
| *mandatory <110% |  |  |

**Figure1**. Population DVH’s for Bladder (a), Small Bowel (b), Large Bowel (c), Left and Right Femoral Heads (d,e) showing the median, interquartile range, min and max values.

a)


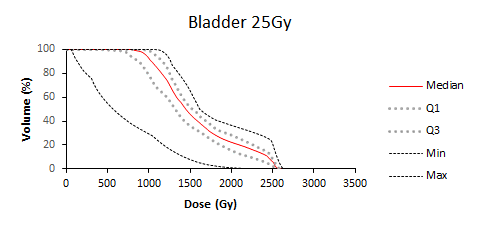

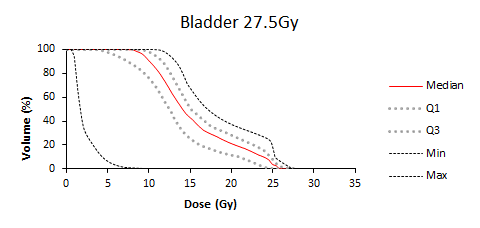


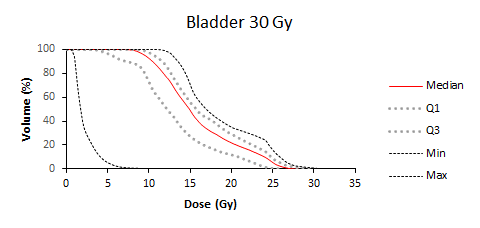

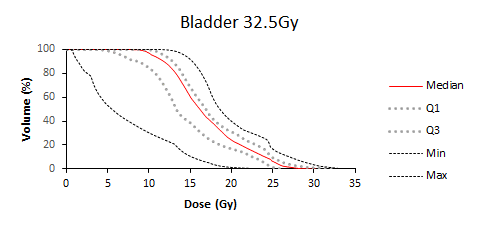


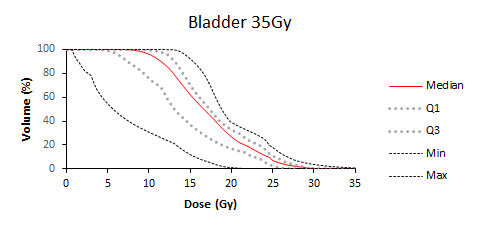


b)


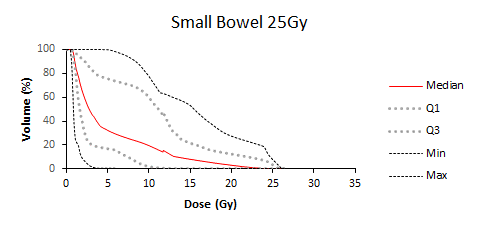

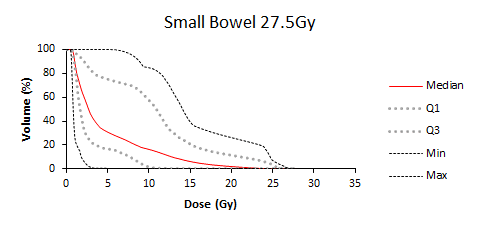


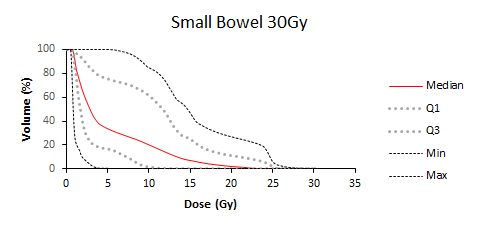

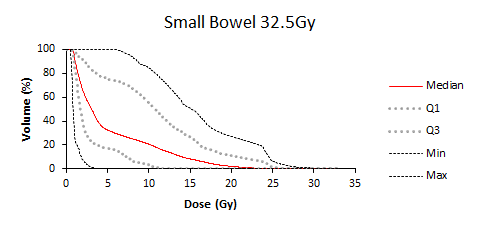


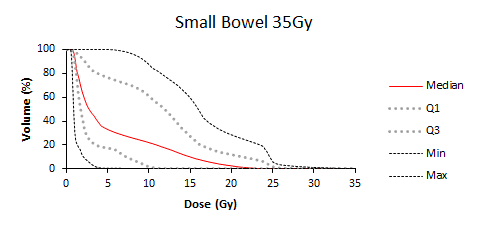


c)


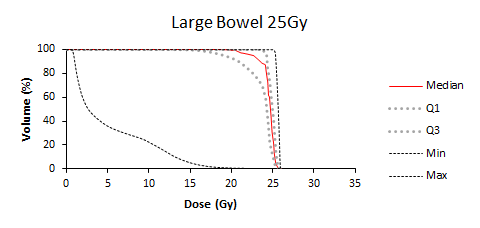

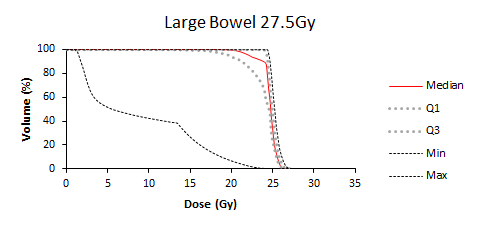

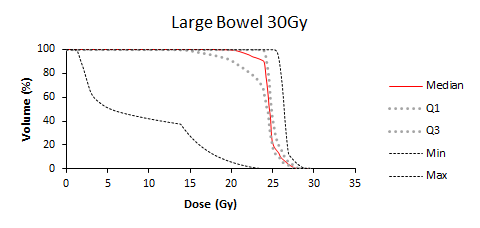

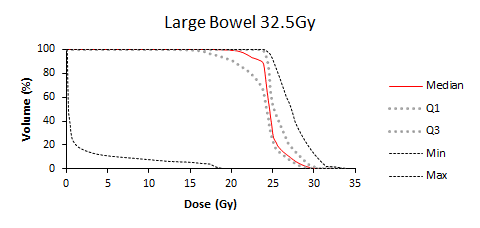


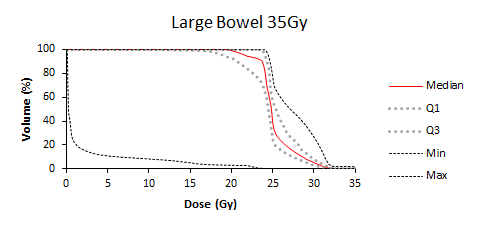


d)


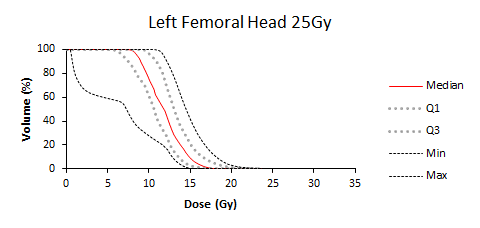

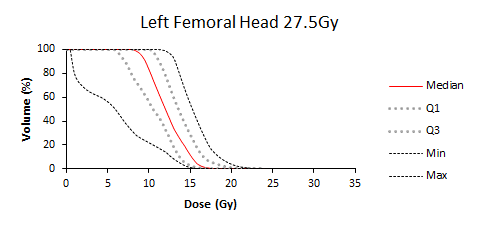


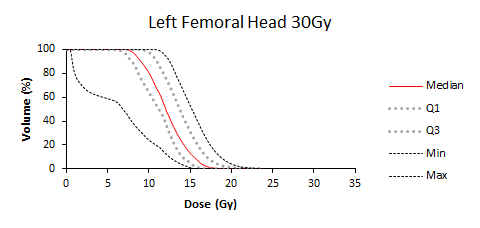

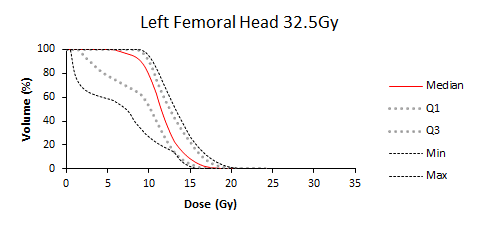


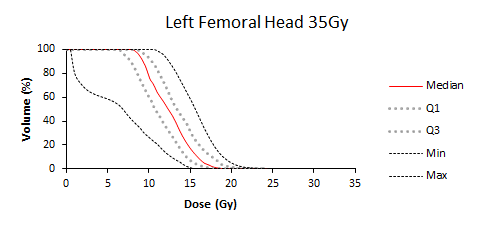


e)


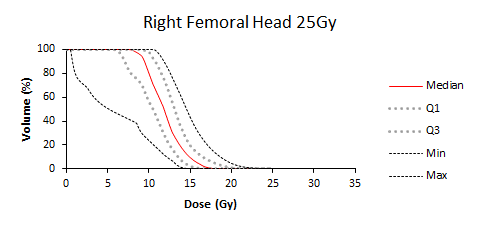

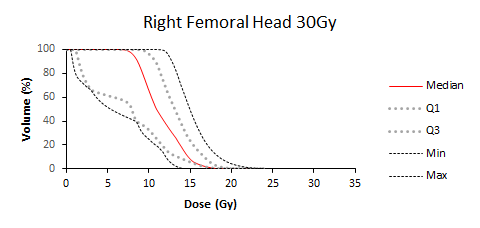

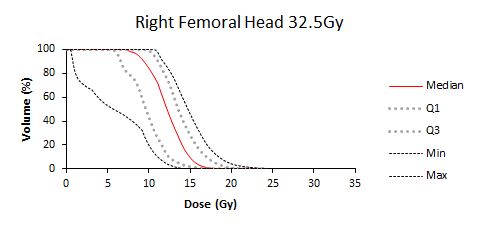


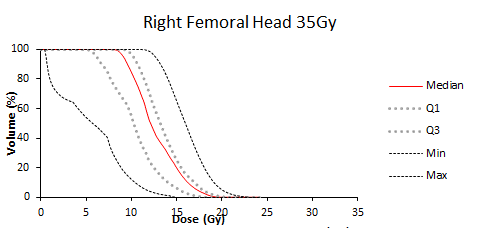


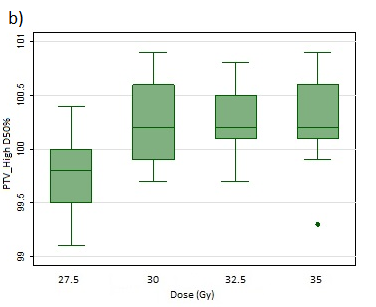
**Figure 2.** Boxplots showing dose constraints at dose levels (27.5Gy, 30Gy, 32.5Gy and 35Gy) for structures 2a) PTV_high D99% > 90, 2b) PTV_high D50%=99-101%, 2c) PTV_high D2% < 105%, 2d) PTV_low D99% >90%, 2e) PTV_low D95% >95%. The band represents the median value the box the first and third quartile. The whiskers show variation above and below this with outliers represented as dots.


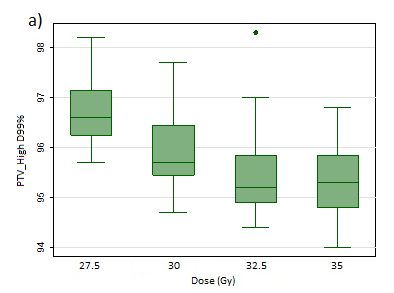


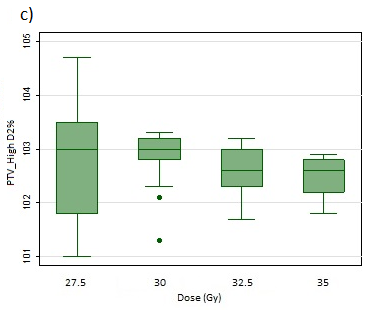


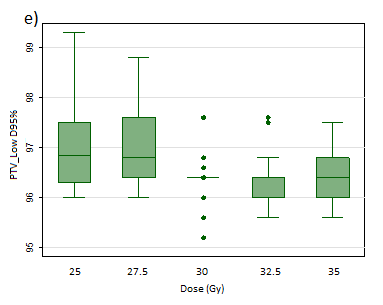

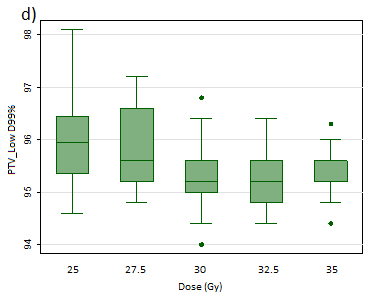


| **Table 2. Dose constraints, mean dose and standard deviation (SD) for structures at 25Gy/5# and each dose level.** | | | | | | | | | | |  |  |
| --- | --- | --- | --- | --- | --- | --- | --- | --- | --- | --- | --- | --- |
|  |  |  |  |  |  |  |  |  |  |  |  |  |
| Structure | | Objective | Standard plan | | Dose level 1 | | Dose level 2 | | Dose level 3 | | Dose level 4 | |
|  |  |  | 25 Gy | | 27.5 Gy | | 30 Gy | | 32.5 Gy | | 35 Gy | |
|  |  |  | Mean | SD | Mean | SD | Mean | SD | Mean | SD | Mean | SD |
| PTVHigh (SIB) | D99% | >90% | ** | ** | 96.7 | 0.7 | 96 | 0.9 | 95.5 | 0.9 | 95.4 | 0.8 |
|  | D95% | >95% | ** | ** | 97.2 | 0.8 | 96.6 | 0.9 | 96.4 | 0.9 | 96.3 | 0.7 |
|  | D50% | 99-101% | ** | ** | 99.8 | 0.4 | 100.2 | 0.4 | 100.3 | 0.3 | 100.3 | 0.4 |
|  | D2% | <105% | ** | ** | 103 | 1 | 102.9 | 0.5 | 102.6 | 0.4 | 102.5 | 0.3 |
| PTVLow | D99% | >90% | 95.9 | 0.8 | 95.8 | 0.7 | 95.3 | 0.6 | 95.3 | 0.6 | 95.2 | 0.6 |
|  | D95% | >95% | 97 | 0.9 | 97 | 0.8 | 96.3 | 0.5 | 96.3 | 0.6 | 96.3 | 0.6 |
|  | D50% | 99-101% * | 99.9 | 0.5 | 100.5 | 0.3 | 100.6 | 0.4 | 100.8 | 0.6 | 101.1 | 1.1 |
| Bladder | D45% | <21Gy | 14.2 | 2.9 | 14 | 3 | 14.4 | 3.1 | 15.4 | 3.2 | 15.7 | 3.7 |
| Small Bowel | D200cc | <20Gy | 3 | 4.9 | 3 | 4.9 | 3.1 | 5.1 | 3.1 | 5 | 3.2 | 5.2 |
|  | D150cc | <22Gy | 4.4 | 5.4 | 4.4 | 5.5 | 4.5 | 5.7 | 4.6 | 5.7 | 4.8 | 6.1 |
|  | D20cc | <25GY | 12.8 | 9.5 | 12.9 | 9.4 | 12.9 | 9.5 | 13 | 9.4 | 13.1 | 9.4 |

**Table 3.** Summary (mean and sd) of the plan quality metrics; total MU, ALPO, MF, average dose-rate, average gantry speed and average total delivery times for all dose level plans.

|  | **25Gy** | **27.5Gy** | **30Gy** | **32.5Gy** | **35Gy** |
| --- | --- | --- | --- | --- | --- |
| **Pass Rate (%) Global** | 99.6 (0.8) | 99.6 (0.5) | 99.5 (0.7) | 99.6 (0.5) | 99.6 (0.4) |
| **Total MU's** | 1443 (178) | 1547 (91) | 1602 (121) | 1700 (119) | 1777 (134) |
| **ALPO** | 4.45 (0.77) | 4.09 (0.46) | 4.00 (0.53) | 3.84 (0.54) | 3.71 (0.47) |
| **MF** | 0.48 (0.06) | 0.49 (0.04) | 0.51 (0.06) | 0.52 (0.07) | 0.53 (0.07) |
| **Average Dose-rate (MU/min)** | 579 (20.4) | 587.7 (8.3) | 595 (3.5) | 598.3 (1.3) | 598.6 (2.1) |
| **Average Gantry Speed (deg/sec)** | 5.01 (0.43) | 4.76 (0.22) | 4.59 (0.3) | 4.36 (0.29) | 4.16 (0.28) |
| **Average Delivery Time (min)** | 2.48 (0.24) | 2.63 (0.16) | 2.69 (0.19) | 2.84 (0.23) | 2.97 (0.22) |
